# Carbon nanotubes as a novel tool for vaccination against infectious diseases and cancer

**DOI:** 10.1186/1477-3155-11-30

**Published:** 2013-09-11

**Authors:** Riccardo Gottardi, Bruno Douradinha

**Affiliations:** 1 Fondazione Ri.MED, Via Bandiera 11, Palermo (PA) 90133, Italy; 2 Department of Orthopaedic Surgery, Center for Cellular and Molecular Engineering, University of Pittsburgh, 450 Technology Drive, Room 239, Pittsburgh (PA) 15219, USA; 3 Department of Chemical and Petroleum Engineering, University of Pittsburgh, 1247 Benedum Hall, 3700 OHara Street, Room 427, Pittsburgh (PA) 15261, USA; 4 University of Pittsburgh Center for Vaccine Research, 3501 Fifth Avenue, BST3 room 9052, Pittsburgh (PA) 15261, USA

**Keywords:** Vaccine, Carbon nanotubes, Functionalization, Infectious diseases, Cancer

## Abstract

Due to their unusual properties, carbon nanotubes have been extensively employed in electronics, nanotechnology and optics, amongst other. More recently, they have also been used as vehicles for drug and antigen delivery, the latter being a novel immunization strategy against infectious diseases and cancer. Here we discuss the potential of carbon nanotubes as an antigen delivery tool and suggest further directions in the field of vaccination.

## Introduction

Carbon nanotubes (CNT) are probably the most famous members of the fullerenes family. Fullerenes comprise any molecule made entirely of carbon atoms, shaped as a sphere, an ellipsoid, or a tube [1]. In 1991, Ijima and colleagues published in *Nature* their description of “helical microtubules of graphitic carbon” [2]. CNT are generally distinguished between single walled carbon nanotubes (SWNT) and multiwall carbon nanotubes (MWNT). The former can be conceptualized as a seamless cylinder obtained by rolling up a single sheet of graphite generally referred to as a graphene layer, i.e., a plane of carbon atoms arranged in a hexagonal lattice. MWNT can be thought as the coaxial assembly of different SWNT of different diameters, one contained within each other. The characteristic aspect ratio of CNT is in the order of millions to one, as their length can span hundreds of microns whereas their diameter is only few nanometers across, and down to less than 0.8 nm for the smaller SWNT. Because of their unique geometry, CNT are often referred to as “one dimensional” [3] and even “zero dimensional” [4] objects.

Their nanometer lateral dimensions are the basis of several of the desirable properties of CNT. However, they also represent a potential limitation, contributing to the inherent difficulties of working with CNT. In fact, pristine nanotubes are virtually insoluble in most solvents in ordinary conditions [5], which limits their large scale processability. Consequently, a large amount of research has been devoted to develop processes that could render CNT soluble, from functionalization of their hydropholbic sidewalls with soluble molecules [6], to wrapping individual CNTs with polymers [7] or DNA [8] and to reduce their length so their dispersibility can be increased [4,9]. Since their popularization in the early ‘90s, CNT have sparked a great deal of excitement in the scientific community and beyond, due to their unique properties, with potential to revolutionize the fields of material science, electronics, energy collection and storage, medicine and many more. CNT have unmatched tensile strength [10], which makes them the strongest material yet discovered, and great thermal conductivity along their axis [11]. In biomedical applications, CNT are mainly investigated either to enhance molecular biosensing [12], due to their optoelectronic properties [13-15], or as drug delivery agents [16]. Since CNT are readily internalized by cells [17], they are ideal vehicles for delivery of therapeutics [18] or diagnostics [19]. CNT can bind macromolecules such as proteins and oligosaccharides, which suggest they would have potential applications as carriers for delivery of active molecules, like drugs or antigens [20-22].

The functionalization of CNT is paramount, first to guarantee the solubility of CNT in aqueous solutions, crucial in a biological context and second, to ensure that the desired molecular agent, either a drug or a marker, is bound to the nanotube. As mentioned above, CNT are poorly soluble in water, and this has initially limited their use as a delivery agent for medicinal purposes [23]. Some approaches were then developed to improve their solubility. The most used is covalent functionalization, in which the desired active molecules bind to cationic functional groups, the latter bound either on the surface or within the walls of CNT. Examples of functional groups are polyethylene glycol (PEG) or ammonium-terminated triethylene glycol, which is a reactive intermediate that allows the synthesis of several functionalized CNT mixtures. Biomolecules such as oligonucleotides and proteins were also used to functionalize CNT. The functional groups are bound to the active molecules, increasing the solubility of CNT [24]. Furthermore, sugar moieties like starch and oligomers like polyvinylpyrrolidone wrap around CNT and transport them to the aqueous phase [7,22]. Both nanotube-mediated oligonucleotide transport inside living cells [25] as well as plasmid DNA gene delivery [26] have shown promising results, which might open the way to the use of CNT as non-viral delivery vectors. The use of CNT carriers could in fact overcome some of the limitations of other non-viral vectors available today, namely the poor pharmacokinetic profiles of the administered oligonucleotide and plasmid DNA conjugates, and the low levels of gene expression obtained [27].

### Carbon nanotubes induce specific and protective Immune responses

An effective vaccine must induce a potent immune response, either at cellular level, stimulating cytotoxic T cells which target and destroy infected cells or at humoral level, through stimulation of the production of neutralizing antibodies which promote opsonization and consequent pathogen clearance. Vaccines against some pathogens, like HIV or Malaria, due to their complexity, would require both responses to be fully efficient in preventing infection and eliminating circulating pathogens. Innate immune responses are also important in vaccination, since they play a crucial role in antigen presentation and immune cells recruitment to infection sites [28].

Several cell types can uptake CNT, including cells of the immune system, such as macrophages, monocytes, natural killer (NK), dendritic cells, T and B cells [17,29-34]. In most cases, CNT did not impair functionality of these cells and it was observed that functionalized CNT are less toxic than pristine CNT, inducing therefore a lesser cytotoxic response [17,35]. CNT were shown to activate cells from the innate immune system, such as monocytes [29,31,34], macrophages [31,32,34] and dendritic cells [30]. Microarray profiling of a monocytic cell line, THP-1, showed that CNT, both functionalized and non-functionalized, activate several genes involved in monocyte response to infection or vaccination, such as nuclear factor kappa-light-chain-enhancer of activated B cells (NF-κB), interleukin-1β (IL-1β), IL-6, tumor necrosis factor-α (TNF-α), among others [29,31]. However, the non-functionalized CNT also increased the expression of genes related to oxidative stress and apoptosis [31]. The functionalized CNT were oxidized and further modified to incorporate ammonium; both versions with and without ammonium were studied, as different diameters (from 9.5 nm to 30 nm). These nanotubes were shown to be non-toxic to both THP-1 cells and human primary monocytes and to induce the production of chemokines in those cells (IL-1β, IL-6,TNF-α and IL-10) [29,35]. These chemokines are involved in many processes, namely recruitment of T cells to infection sites and inflammation. Interestingly, CNT of low diameter (9.5 nm) and lacking the ammonium group failed in activating the pathways above mentioned and also in inducing production of chemokines in monocytes [29]. These studies suggest that CNT are able to induce an innate immune response dependent both on their functionalization type and size. Macrophages derived from THP-1 cells presented the same phenotype as just described for monocytes [31].

Antigen presentation is a process crucial to mount an immune response against foreign antigens or tumor antigens. Professional antigen presenting cells (APC), such as dendritic cells or macrophages, will uptake a source of antigen (a microorganism, an infected or tumor cell, or a vaccine carrier, such as CNT), degrade it and present it to T cells through the surface complexes MHC class I or class II. While MHC class I induces a more cytotoxic CD8^+^ T cell response, MHC class II shifts the immune response to helper CD4^+^ T cells which will promote an antibody based response [28]. CNT activate both MHC class I and class II, the latter to a greater extent [30,36], suggesting they will induce preferably a humoral response. CNT carrying peptides of immunogens of some pathogens or tumors have been shown to be immunogenic and protective in experimental animal models. CNT containing either peptides for a B cell epitope from the foot-and-mouth disease virus (FMDV), which causes huge economic losses in the cattle industry [37], or the N-terminal (residues 21–42) of *Plasmodium vivax* Apical Membrane Antigen-1 (AMA-1, a micronemal protein highly conserved amongst *Plasmodium* species, the causative agent of Malaria [28,38]), induced a high titer of antibodies [23,39]. Further tests confirmed that the antibodies generated were specific for the regions present in the CNT. They were able to fully neutralize the FMDV infection in vitro and some of the animals immunized with the *Plasmodium vivax* AMA-1 N-terminal were fully protected against challenge with a murine plasmodial species, *Plasmodium berghei*, both sharing a high homology for that AMA-1 region [23,39]. A very recent work showed that a peptide of Wilm’s tumor protein 1 (WT1), a kidney cancer, conjugated to SWNT is rapidly internalized by human APC, namely dendritic cells and macrophages [30]. The SWNT conjugated WT1 peptides were internalized through a macropinocytosis mechanism, known to uptake several macromolecular antigens in immature dendritic cells. They were found to co-localize in lysomal compartments, which lead to the degradation of antigens and promote their presentation to the immune system through MHC class II molecules. Both conjugated and unconjugated WT1 peptide elicited a similar CD4^+^ T cell interferon-γ (IFN-γ) dependent response in human lymphocytes. However, SWNT conjugated peptides induced a much higher humoral response in Balb/c mice when TiterMax, a commercial adjuvant, was used. This is of particular importance, since tumor self-antigens usually induce poor immune responses.

As mentioned above, CNT can also activate MHC class I receptors [36], which would induce a cytotoxic, Th1 biased cell response, characterized by the production of cytokines such as IFN-γ, TNF-α and IL-12. Although pristine CNT do not induce a CD8^+^ T cell response, there is indirect evidence that functionalized, conjugated SWNT and MWNT originate such response. Zeinali and colleagues tested SWNT coated with tuberculin purified protein derivative (PPD), a mixture of antigens derived from a culture of *Mycobacterium tuberculosis*, the pathogen responsible for tuberculosis [40]. Balb/c mice were immunized with tuberculin PPD, either free or bound to SWNT, receiving two immunizations, 2-weeks apart. Two groups were immunized with free tuberculin PPD, one adjuvanted with complete Freund’s adjuvant (CFA) while the other received no adjuvant. The authors did not assess protection against challenge with an infectious bacillus, but compared the cytokine profile induced by the immunization with tuberculin, free or attached to SWNT, with the one elicited by immunization with BCG (a tuberculosis vaccine based on attenuated *Mycobacterium bovis*). The levels of IFN-γ, IL-12, IL-5 and IL-10 were measured in the cultures of splenocytes isolated from the immunized mice. Both groups immunized with BCG and tuberculin PPD bound to SWNT had significantly higher levels of IFN-γ when compared to mice immunized with free tuberculin, adjuvanted or not with CFA. Conversely, the latter groups had significantly higher levels of Th2-type cytokines (IL-5 and IL-10) than the BCG and SWNT immunized groups. Another work shows evidence of cytotoxic T cells induced by CNT. Meng and colleagues conjugated proteins from cellular lysates derived from the murine H22 liver cancer to previously oxidized MWNT [41]. Balb/c mice immunized with a tumor cell vaccine (TCV) based on the H22 liver cancer cells and with MWNT containing proteins of the same cells had a higher cure rate than those immunized with just TCV or TCV together with non-conjugated nanotubes. Also, immunized mice were protected upon further challenge with H22 liver cancer, never developing cancer unlike control mice. Furthermore, the protection observed was specific, since mice challenged with a different tumor cell line were not protected. Moreover, lymphocytes isolated from immunized mice targeted H22 liver cells, but not the other tumor cell line.

Overall, more work is required to fully understand the action of CNT on the immune system. However, the works described above show clearly that CNT activate the innate immune response, as observed by the transcription of genes involved in several pathways of inflammation, response to infection and vaccination and release of chemokines which will attract APC and consequent mount an adaptive immune response. Macrophages and dendritic cells process efficiently peptides incorporated in CNT and present them in MHC Class I and II, promoting preferably a humoral response against antigens conjugated to them, but also a cellular immune response. CNT were not immunogenic by themselves, since no specific immune response for them was observed [29,30], indicating they can be used when regimens of multiple immunizations are required without risk of losing their stimulatory properties.

### Carbon nanotubes as potential adjuvants

Many vaccines use adjuvants, substances that enhance the immunogenic potential of the immunization strategy, without inducing an immune response per se [28]. As described above, CNT stimulate the innate immune system, therefore having inherent adjuvant properties [29,31]. Another work showed that, when administration of embryonic stem cells (ESC) is adjuvanted with MWNT, the therapeutic effect of the former in a colon cancer C57Bl/6 mouse model is enhanced [42], promoting a decrease in the tumor volumes and an increase in cytotoxic CD8^+^ T cells and Th1-type cytokines, such as IFN-γ and IL-2.

It has been suggested that SWNT conjugated with unmethylated CpG DNA motifs can be used as an adjuvant in vaccines [43]. These DNA motifs can be considered a danger signal by the immune system, recognized by Toll-like receptor 9 (TLR-9), an endocytic receptor, and confer protection against several intracellular pathogens and tumors by improving the immune response against them. However, the action of the sole CpG DNA motifs is short-lived and requires administration of high and constant doses, due to the fact both CpG and cellular membranes have a negative charge, impairing the uptake of the former by the cells. Bianco and colleagues conjugated a CpG motif in SWNT, improving their immunostimulatory properties. When incubated with mouse splenocytes, CpG 1668 conjugated to SWNT leads to a decrease in the production of IL-6, compared with the non-conjugated form. Since this cytokine is pro-inflammatory, the authors believe administration of CpG conjugated with SWNT would be less toxic than using the CpG alone. Also, the SWNT would compensate for the negative charge of the CpG, facilitating its entry in the cells. Another work showed that CpG conjugated with functionalized SWNTs (CpG-SWNT) were avidly internalized by immune cells in a mouse brain tumor (gliomas) model [34]. Unlike the previous work, a pro-inflammatory cytokine response was observed, as shown by the increase in the production of IL-12 and TNF-α by monocytes which suffered uptake of CpG-SWNT. NK cells, macrophages and microglia (APC resident in brain vasculature) also readily uptake CpG-SWNT, resulting in eradication of gliomas in more than 50% of the animals and long lasting immunity when cured mice were re-challenged with homologous tumor. Protection was dependent on NK and CD8^+^ T cells. Neither administration of CpG alone nor CpG co-administrated with unconjugated CNT mimicked these results. This work illustrates the potential of CNT to deliver adjuvant molecules (in this case, CpG) in a mouse model of gliomas, clearly contributing to clearance and immunization against this tumor, and suggests the use of this strategy as adjuvants either for other tumors or even for intracellular pathogens.

### Toxicity of carbon nanotubes

One of the main concerns regarding the use of CNT in vaccines and other human therapeutic or prophylactic interventions is their toxicity. Due to their small size, they can spread within the organism, reaching several crucial sites, which represents both a concern in terms of cytoxicity and an opportunity in terms of their potential as vaccine carriers. Several evidences show that CNT can be toxic both *in vitro* and *in vivo*, inducing the production of cytotoxic reactive oxygen species (ROS), cell apoptosis and necrosis [24,44,45]. It has also been shown that CNT can bind to several plasma proteins and can activate the innate immune system complement pathways, leading to inflammation [46]. Although this could have an adjuvant effect, in excess it could be deleterious for the host if high levels of inflammation are induced. However, the toxicity of CNT is dependent of several factors, namely the dose used and their solubility in water. Unsurprisingly, the higher the dose, the more pronounced are the toxic effects. It could in fact be argued that for most compounds, very high doses induce a toxic effect. Furthermore, CNT that are functionalized in order to be more soluble in water are less toxic [16]. In particular, PEG or PEG-phospholipid dispersed CNT were shown to be well tolerated in biological systems both in vivo and in vitro [44,47]. Functionalized CNT are also more biocompatible in mice, being slowly excreted through urine and feces, therefore being less prone to potential toxic effects caused by accumulation, and are also more hemocompatible, since hematological analyses show no major differences between naïve animals and mice subjected to CNT [44,45,48,49]. Additionally, an accurate purification of CNT to ensure toxic chemicals or metals used during their production and in the functionalization steps are effectively removed, also reduces their toxicity. However, this process is very laborious and complex. Overall, the actual level of toxicity of CNT, as shown in the literature and assessed by our own experience, remains controversial and studies with established toxicology models have still to be optimized and implemented [16].

It is important to note that the majority of the works mentioned above, do not report any adverse effect of CNT in cells or mice, including those that specifically addressed the issue of toxicity. For the tuberculin PPD study, Zeinali and colleagues showed that although SWNT generate ROS in Balb/c mice splenocytes, it had no effect in the cell viability [40]. Also, nitric oxide, an end product of inducible nitric oxide synthase, which would be an indication of an inflammatory response, could not be detected in the supernatants of mouse macrophage cultures previously incubated with SWNT alone or conjugated with PPD. Moreover, immature dendritic cells incubated with doses of SWNT up to 100 μg/mL were not affected in their functionality and viability [30].

Toxicity also varies according to the different routes of administration [44,45]. Non-dispersed CNT tend to form larger aggregates and therefore are more toxic. Works with pristine CNT administered through the respiratory tract in animal models show a high degree of inflammation, granuloma formation and obstruction of the upper airways [31,45,50]. Even functionalized CNT seem to induce toxicity in lungs in rats [51]. Although some strategies were shown to circumvent the issue of toxicity following intranasal administration, such as CN_X_ NT, nanotubes doped with nitrogen groups [52], the risk of mechanical obstruction may persist, making this route less appealing for immunization with CNT. Administration of pristine CNT subcutaneously and intraperitoneally in mice also led to undesired toxic effects, such as granuloma formation or inflammation [45,53,54]. Conversely, when CNT which suffered several functionalization methods (e.g., PEG, amino acids, etc.) were injected in mice using the same routes, only in some cases low levels of inflammation were observed [23,30,39-41,44,55,56]. Oral administration of both pristine and functionalized CNT did not induce toxicity in mice [44,45,53,57,58].

An useful overview of the concerns related to toxicity in the biomedical applications of nanotubes and the related regulatory issues has been elegantly discussed elsewhere [59].

## Conclusions

It is clear that CNT have a high potential for delivery of antigens and to be considered as a novel vaccine platform for both infectious diseases and cancer, due to the promising results mentioned above. Their nanometric dimension allows them to be easily internalized by cells. They have a large inner volume compared to their linear dimensions and biomolecules can be easily immobilized on their outer surface. Such confers them an advantage to be used as nanocarriers for controlled and targeted drug delivery [59]. As stated, further studies regarding the toxicity of these nanostructures are required and new approaches are being undertaken to enhance the capacity of cells to degrade CNT, such as shortening of CNT [4]. Their immunization potential can be improved upon confirmation that the conjugated epitopes maintain their correct conformation, a key aspect for an efficient immune response elicited against them [60]. Furthermore, the recent discovery that certain enzymes such as myeloperoxidases can degrade CNT leads to believe that, if antigens are encapsulated within these nanostructures, they would be more protected from external factors [61]. After cellular internalization and degradation of CNT, antigens could still be efficiently presented. Functionalized CNT can be administered using routes commonly used in vaccination, such as subcutaneous and oral without inducing severe, undesired toxic effects [23,30,39-41,44,55-57] which strengthens the possibility of their usefulness as immunization strategies.

Nevertheless, the promise of CNT to revolutionize the field of biomedical applications is yet unmet. In fact, as clearly described by Kostarelos and colleagues “The use of CNT in medicine is now at the crossroads between a proof-of-principle concept and an established preclinical candidate for a variety of therapeutic and diagnostic applications. Progress towards clinical trials will depend on the outcomes of efficacy and toxicology studies, which will provide the necessary risk-to-benefit assessments for carbon nanotube based materials” [16]. Whether CNT will meet these expectations is at the moment difficult to predict, but everything indicates most probably they will. Not long ago, the use of CNT in material science applications was even regarded as some science fiction tool [62]. However, nowadays, yarns of nanotubes are routinely produced in an up scalable process [63], and can be woven to form ropes and fabrics with exceptional properties [64]. Current and further research will definitely unravel the potential of CNT to be used in vaccination approaches against infectious diseases and cancer.

## Abbreviations

CNT: Carbon nanotubes; SWNT: Single walled carbon nanotubes; MWNT: Multiwall carbon nanotubes; DNA: Deoxyribonucleic acid; PEG: Polyethylene glycol; IFN-γ: Interferon-α; IL: Interleukin; TNF-α: Tumor necrosis factor α; NF-κB: Nuclear factor kappa-light-chain-enhancer of activated B cells; FMDV: Foot-and-mouth disease virus; CFA: Complete Freund’s Adjuvant; iCFA: Incomplete Freund’s Adjuvant; NK: Natural killer cells; AMA-1: Apical membrane antigen-1; BCG: Bacillus calmette-guerin; PPD: Purified protein derivative; ESC: Embryonic stem cells; TCV: Tumor cell vaccine; WT1: Wilm’s tumor protein 1; APC: Antigen presenting cell; ROS: Reactive oxygen species.

## Competing interests

The authors declare that they have no competing interests.

## Authors’ contributions

RG and BD participated equally in the drafting and writing of this manuscript. Both authors read and approved the final manuscript.

## References

[B1] Schultz HP Topological Organic Chemistry. Polyhedranes and Prismanes J Org Chem 1965 30 1361 1364 10.1021/jo01016a005

[B2] Iijima S Helical microtubules of graphitic carbon Nature 1991 354 56 58 10.1038/354056a0

[B3] Geim AK Novoselov KS The rise of graphene Nat Mat 2007 6 183 191 10.1038/nmat1849 17330084

[B4] Kamalasanan K Gottardi R Tan S Chen Y Godugu B Rothstein S Balazs AC Star A Little SR “Zero-Dimensional” Single-Walled Carbon Nanotubes Angew Chem Int Ed Engl 2013 10.1002/anie.201305526 24038731

[B5] Davis VA Parra-Vasquez ANG Green MJ Rai PK Behabtu N Prieto V Booker RD Schmidt J Kesselman E Zhou W Fan H Adams WW Hauge RH Fischer JE Cohen Y Talmon Y Smalley RE Pasquali M True solutions of single-walled carbon nanotubes for assembly into macroscopic materials Nat Nanotecnol 2009 4 830 834 10.1038/nnano.2009.302 19893518

[B6] Balasubramanian K Burghard M Chemically functionalized carbon nanotubes Small 2005 1 180 192 10.1002/smll.200400118 17193428

[B7] O’Connell MJ Boul P Ericson LM Huffman C Wang Y Haroz E Kuper C Tour J Ausman KD Smalley RE Reversible water-solubilization of single-walled carbon nanotubes by polymer wrapping Chem Phys Letters 2001 342 265 271 10.1016/S0009-2614(01)00490-0

[B8] Zheng M Jagota A Semke ED Diner BA McLean RS Lustig SR Richardson RE Tassi NG DNA-assisted dispersion and separation of carbon nanotubes Nat Mat 2003 2 338 342 10.1038/nmat877 12692536

[B9] Chen Z Kobashi K Rauwald U Booker R Fan H Hwang W-F Tour JM Soluble ultra-short single-walled carbon nanotubes J Am Chem Soc 2006 128 10568 10571 10.1021/ja063283p 16895425

[B10] Yu M-F Lourie O Dyer MJ Moloni K Kelly TF Ruoff RS Strength and Breaking Mechanism of Multiwalled Carbon Nanotubes Under Tensile Load Science 2000 287 637 640 10.1126/science.287.5453.637 10649994

[B11] Pop E Mann D Wang Q Goodson K Dai H Thermal conductance of an individual single-wall carbon nanotube above room temperature Nano Lett 2006 6 96 100 10.1021/nl052145f 16402794

[B12] Yang W Thordarson P Gooding JJ Ringer SP Braet F Carbon nanotubes for biological and biomedical applications Nanotechnology 2007 18 412001 10.1088/0957-4484/18/41/412001

[B13] Wang J Carbon-Nanotube Based Electrochemical Biosensors: A Review Electroanalysis 2005 17 7 14 10.1002/elan.200403113

[B14] Cai D Ren L Zhao H Xu C Zhang L Yu Y Wang H Lan Y Roberts MF Chuang JH Naughton MJ Ren Z Chiles TC A molecular-imprint nanosensor for ultrasensitive detection of proteins Nat Nanotecnol 2010 5 597 601 10.1038/nnano.2010.114 PMC3064708 20581835

[B15] Heller DA Jin H Martinez BM Patel D Miller BM Yeung T-K Jena PV Höbartner C Ha T Silverman SK Strano MS Multimodal optical sensing and analyte specificity using single-walled carbon nanotubes Nat Nanotecnol 2009 4 114 120 10.1038/nnano.2008.369 19197314

[B16] Kostarelos K Bianco A Prato M Promises, facts and challenges for carbon nanotubes in imaging and therapeutics Nat Nanotecnol 2009 4 627 633 10.1038/nnano.2009.241 19809452

[B17] Kostarelos K Lacerda L Pastorin G Wu W Wieckowski S Luangsivilay J Godefroy S Pantarotto D Briand J-P Muller S Prato M Bianco A Cellular uptake of functionalized carbon nanotubes is independent of functional group and cell type Nat Nanotecnol 2007 2 108 113 10.1038/nnano.2006.209 18654229

[B18] Liu Z Chen K Davis C Sherlock S Cao Q Chen X Dai H Drug delivery with carbon nanotubes for in vivo cancer treatment Cancer Res 2008 68 6652 6660 10.1158/0008-5472.CAN-08-1468 18701489 PMC2562710

[B19] Welsher K Liu Z Sherlock SP Robinson JT Chen Z Daranciang D Dai H A route to brightly fluorescent carbon nanotubes for near-infrared imaging in mice Nat Nanotecnol 2009 4 773 780 10.1038/nnano.2009.294 PMC2834239 19893526

[B20] Balavoine F Schultz P Richard C Mallouh V Ebbesen TW Mioskowski C Helical Crystallization of Proteins on Carbon Nanotubes: A First Step towards the Development of New Biosensors Angew Chem Int Ed Engl 1999 38 1912 1915 10.1002/(SICI)1521-3773(19990712)38:13/14<1912::AID-ANIE1912>3.0.CO;2-2 34182705

[B21] Chen RJ Zhang Y Wang D Dai H Noncovalent sidewall functionalization of single-walled carbon nanotubes for protein immobilization J Am Chem Soc 2001 123 3838 3839 10.1021/ja010172b 11457124

[B22] Star A Steuerman DW Heath JR Stoddart JF Starched carbon nanotubes Angew Chem Int Ed Engl 2002 41 2508 2512 10.1002/1521-3773(20020715)41:14<2508::AID-ANIE2508>3.0.CO;2-A 12203517

[B23] Pantarotto D Partidos CD Graff R Hoebeke J Briand J Prato M Bianco A Synthesis , Structural Characterization, and Immunological Properties of Carbon Nanotubes Functionalized with Peptides J Am Chem Soc 2003 125 6160 6164 10.1021/ja034342r 12785847

[B24] Foldvari M Bagonluri M Carbon nanotubes as functional excipients for nanomedicines: II. Drug delivery and biocompatibility issues Nanomedicine 2008 4 183 200 10.1016/j.nano.2008.04.003 18550450

[B25] Liu Z Winters M Holodniy M Dai H siRNA delivery into human T cells and primary cells with carbon-nanotube transporters Angew Chem Int Ed Engl 2007 46 2023 2027 10.1002/anie.200604295 17290476

[B26] Pantarotto D Singh R McCarthy D Erhardt M Briand J-P Prato M Kostarelos K Bianco A Functionalized carbon nanotubes for plasmid DNA gene delivery Angew Chem Int Ed Engl 2004 43 5242 5246 10.1002/anie.200460437 15455428

[B27] Niidome T Huang L Gene therapy progress and prospects: nonviral vectors Gene Ther 2002 9 1647 1652 10.1038/sj.gt.3301923 12457277

[B28] Douradinha B Doolan DL Harnessing immune responses against Plasmodium for rational vaccine design Trends Parasitol 2011 27 274 283 10.1016/j.pt.2011.01.002 21531627

[B29] Pescatori M Bedognetti D Venturelli E Ménard-moyon C Bernardini C Muresu E Piana A Maida G Manetti R Sgarrella F Bianco A Gemma L Biomaterials Functionalized carbon nanotubes as immunomodulator systems Biomaterials 2013 34 4395 4403 10.1016/j.biomaterials.2013.02.052 23507086

[B30] Villa CH Dao T Ahearn I Fehrenbacher N Casey E Rey D Korontsvit T Zakhaleva V Batt C Philips MR Scheinberg D Single-walled carbon nanotubes deliver peptide antigen into dendritic cells and enhance IgG responses to tumor-associated antigens ACS Nano 2011 5 5300 5311 10.1021/nn200182x 21682329 PMC3143710

[B31] Chou C-C Hsiao H-Y Hong Q-S Chen C-H Peng Y-W Chen H-W Yang P-C Single-walled carbon nanotubes can induce pulmonary injury in mouse model Nano Lett 2008 8 437 445 10.1021/nl0723634 18225938

[B32] Fiorito S Serafino A Andreola F Bernier P Effects of fullerenes and single-wall carbon nanotubes on murine and human macrophages Carbon 2006 44 1100 1105 10.1016/j.carbon.2005.11.009

[B33] Pulskamp K Diabat S Krug HF Carbon nanotubes show no sign of acute toxicity but induce intracellular reactive oxygen species in dependence on contaminants Toxicol Lett 2007 168 58 74 10.1016/j.toxlet.2006.11.001 17141434

[B34] Zhao D Alizadeh D Zhang L Carbon Nanotubes Enhance CpG Uptake and Potentiate Antiglioma Immunity Clin Cancer Res 2011 17 771 782 10.1158/1078-0432.CCR-10-2444 21088258 PMC3041854

[B35] Delogu LG Venturelli E Manetti R Carru C Madeddu R Murgia L Sgarrella F Bianco A Ex vivo impact of functionalized carbon nanotubes on human immune cells Nanomedicine 2012 7 231 243 10.2217/nnm.11.101 22106855

[B36] Koyama S Endo M Kim Y-A Hayashi T Yanagisawa T Osaka K Koyama H Haniu H Kuroiwa N Role of systemic T-cells and histopathological aspects after subcutaneous implantation of various carbon nanotubes in mice Carbon 2006 44 1079 1092 10.1016/j.carbon.2005.08.006

[B37] Rodriguez LL Gay CG Development of vaccines toward the global control and eradication of foot-and-mouth disease Expert Rev Vaccines 2011 10 377 387 10.1586/erv.11.4 21434805

[B38] Remarque EJ Faber BW Kocken CHM Thomas AW Apical membrane antigen 1: a malaria vaccine candidate in review Trends Parasitol 2008 24 74 84 10.1016/j.pt.2007.12.002 18226584

[B39] Yandar N Pastorin G Prato M Bianco A Patarroyo ME Manuel Lozano J Immunological profile of a Plasmodium vivax AMA-1 N-terminus peptide-carbon nanotube conjugate in an infected Plasmodium berghei mouse model Vaccine 2008 26 5864 5873 10.1016/j.vaccine.2008.08.014 18771700

[B40] Zeinali M Jammalan M Ardestani SK Mosaveri N Immunological and cytotoxicological characterization of tuberculin purified protein derivative (PPD) conjugated to single-walled carbon nanotubes Immunol Lett 2009 126 48 53 10.1016/j.imlet.2009.07.012 19664657

[B41] Meng J Duan J Kong H Li L Wang C Xie S Chen S Gu N Xu H Yang X-D Carbon nanotubes conjugated to tumor lysate protein enhance the efficacy of an antitumor immunotherapy Small 2008 4 1364 1370 10.1002/smll.200701059 18720440

[B42] Mocan T Iancu C Effective colon cancer prophylaxis in mice using embryonic stem cells and carbon nanotubes Int J Nanomedicine 2011 6 1945 1954 21976971 10.2147/IJN.S24060 PMC3181055

[B43] Bianco A Hoebeke J Godefroy S Chaloin O Pantarotto D Briand J-P Muller S Prato M Partidos CD Cationic carbon nanotubes bind to CpG oligodeoxynucleotides and enhance their immunostimulatory properties J Am Chem Soc 2005 127 58 59 10.1021/ja044293y 15631447

[B44] Yang S Luo J Zhou Q Wang H Pharmacokinetics, Metabolism and Toxicity of Carbon Nanotubes for Bio- medical Purposes Theranostics 2012 2 271 282 10.7150/thno.3618 22509195 PMC3326738

[B45] Kolosnjaj-Tabi J Szwarc H Moussa F Hashim AA In vivo Toxicity Studies of Pristine Carbon Nanotubes: A Review The Delivery of Nanoparticles. Volume 2000 2012 Rijeka, Croatia: InTech 37 58

[B46] Salvador-Morales C Flahaut E Sim E Sloan J Green MLH Sim RB Complement activation and protein adsorption by carbon nanotubes Mol Immunol 2006 43 193 201 10.1016/j.molimm.2005.02.006 16199256

[B47] Liu Z Davis C Cai W He L Chen X Dai H Circulation and long-term fate of functionalized, biocompatible single-walled carbon nanotubes in mice probed by Raman spectroscopy Proc Natl Acad Sci U S A 2008 105 1410 1415 10.1073/pnas.0707654105 18230737 PMC2234157

[B48] Lacerda L Ali-Boucetta H Herrero M Pastorin G BiancoA G Prato M Kostarelos K Tissue histology and physiology following intravenous administration of different types of functionalized multiwalled carbon nanotubes Nanomedicine 2008 3 149 161 10.2217/17435889.3.2.149 18373422

[B49] Schipper ML Nakayama-Ratchford N Davis CR Kam NWS Chu P Liu Z Sun X Dai H Gambhir SS A pilot toxicology study of single-walled carbon nanotubes in a small sample of mice Nat Nanotecnol 2008 3 216 221 10.1038/nnano.2008.68 18654506

[B50] Yu Y Zhang Q Mu Q Zhang B Yan B Exploring the immunotoxicity of carbon nanotubes Nanoscale Res Lett 2008 3 271 277 10.1007/s11671-008-9153-1 21771349 PMC3244872

[B51] Coccini T Manzo L Roda E Safety evaluation of engineered nanomaterials for health risk assessment: an experimental tiered testing approach using pristine and functionalized carbon nanotubes ISRN Toxicol 2013 2013 1 13 10.1155/2013/825427 PMC3658371 23724301

[B52] Carrero-Sanchez JC Elías aL Mancilla R Arrellín G Terrones H Laclette JP Terrones M Biocompatibility and toxicological studies of carbon nanotubes doped with nitrogen Nano Lett 2006 6 1609 1616 10.1021/nl060548p 16895344

[B53] Kolosnjaj-tabi J Hartman KB Boudjemaa S Ananta JS Morgant G Szwarc H Wilson LJ Moussa F In Vivo Behavior of Large Doses of Walled Carbon Nanotubes after Oral and Intraperitoneal Administration to Swiss Mice ACS Nano 2010 4 1481 1492 10.1021/nn901573w 20175510

[B54] Poland C Duffin R Kinloch I Maynard A Wallace WH Seaton A Stone V Brown S Macnee W Donaldson W Carbon nanotubes introduced into the abdominal cavity of mice show asbestos-like pathogenicity in a pilot study Nat Nanotecnol 2008 3 423 428 10.1038/nnano.2008.111 18654567

[B55] Bellucci S Chiaretti M Cucina A Carru G Chiaretti aI Multiwalled carbon nanotube buckypaper: toxicology and biological effects in vitro and in vivo Nanomedicine 2009 4 531 540 10.2217/nnm.09.36 19572819

[B56] Pantarotto D Partidos CD Hoebeke J Brown F Kramer E Briand J Muller S Prato M Bianco A Immunization with Peptide-Functionalized Carbon Nanotubes Enhances Virus-Specific Neutralizing Antibody Responses Chem Biol 2003 10 961 966 10.1016/j.chembiol.2003.09.011 14583262

[B57] Folkmann JK Risom L Jacobsen NR Wallin H Loft S Møller P Oxidatively damaged DNA in rats exposed by oral gavage to C60 fullerenes and single-walled carbon nanotubes Environ Health Perspect 2009 117 703 7038 19479010 10.1289/ehp.11922 PMC2685830

[B58] Lim J-H Kim S-H Shin I-S Park N-H Moon C Kang S-S Kim S-H Park S-C Kim J-C Maternal exposure to multi-wall carbon nanotubes does not induce embryo-fetal developmental toxicity in rats Birth Defects Res B Dev Reprod Toxicol 2011 92 69 76 10.1002/bdrb.20283 21254368

[B59] Beg S Rizwan M Sheikh AM Hasnain MS Anwer K Kohli K Advancement in carbon nanotubes: basics, biomedical applications and toxicity J Pharm Pharmacol 2011 63 141 163 10.1111/j.2042-7158.2010.01167.x 21235578

[B60] Purcell AW McCluskey J Rossjohn J More than one reason to rethink the use of peptides in vaccine design Nat Rev Drug Disc 2007 6 404 414 10.1038/nrd2224 17473845

[B61] Kagan VE Konduru NV Feng W Allen BL Conroy J Volkov Y Vlasova II Belikova NA Yanamala N Kapralov A Tyurina YY Shi J Kisin ER Murray AR Franks J Stolz D Gou P Klein-Seetharaman J Fadeel B Star A Shvedova AA Carbon nanotubes degraded by neutrophil myeloperoxidase induce less pulmonary inflammation Nat Nanotecnol 2010 5 354 359 10.1038/nnano.2010.44 PMC6714564 20364135

[B62] Collins PG Avouris P Nanotubes for Electronics Sci Am 2000 12 62 69 11103460 10.1038/scientificamerican1200-62

[B63] Ericson LM Fan H Peng H Davis V Zhou W Sulpizio J Wang Y Booker R Vavro J Guthy C Parra-Vasquez aNG Kim MJ Ramesh S Saini RK Kittrell C Lavin G Schmidt H Adams WW Billups WE Pasquali M Hwang W-F Hauge RH Fischer JE Smalley RE Macroscopic, neat, single-walled carbon nanotube fibers Science 2004 305 1447 1450 10.1126/science.1101398 15353797

[B64] Behabtu N Green M Pasquali M Carbon nanotube-based neat fibers Nano Today 2008 3 24 34 10.1016/S1748-0132(08)70062-8

